# Supplementary material for: Altered Clock Gene Expression in Female APP/PS1 Mice and Aquaporin-Dependent Amyloid Accumulation in the Retina
Source: Int J Mol Sci. 2023 Oct 27;24(21):15679. doi: 10.3390/ijms242115679 (PMC10648501; doi:10.3390/ijms242115679)
Supplement: Supplementary file 1 [file ijms-24-15679-s001.zip › ijms-2663697-supplementary.pdf]

Supplementary Table S1. Real-time PCR oligonucleotides.

Gene	Sequence (5´>3´)	
	Forward	Reverse
Clock	ccttgcgtctgtgggtgtt	tgtcatcttcttccaccaatcca
Arntl	cgcctctacctgttcaaagaaaaa	tcacccgtatttccccgttc
Per1	aaacggcaagcggatgg	gccatacagtggaggacgaaa
Per2	cagaggagaagactccgcac	ttgctgtcgctggatgatgt
Per3	acgaagctcctcgaatccct	gaatctgacgggcgagtgtt
Cry1	cggtagaggaagtcggggtg	tgaagaactgaccaagcggg
Cry2	tgaagaactgaccaagcggg	gttggtgattggcttctctgc
Hprt	gttgggcttacctcactgct	taatcacgacgctgggactg
AQP0	cgtgctctgcatctttgcta	accctccccacagtctcttt
Aqp1	tcccctaactttcccctttg	agcacagggacaattccaag
Aqp3	cttgtgatgtttggctgtgg	aagccaagttgatggtgagg
Aqp4	ttccgttcgatcttcagagg	tatcagcccatttcccagag
Aqp5	ttcaggaccatcccagaaag	taagatggcactcgacgaac
AQP7	atatgtgctgggtcagttcctg	aatgtttgcagtggccttgg
Aqp8	ttgctaccttggggaacatc	caatcagccctccaaatagc
Aqp9	tgcgacttttggtgtctctg	ttgaaccactccatccttcc
AQP11	ctgctggctgcactcatc	ttgagaaatacaggctac
Gs	actgtgagcccaagtgtgtg	ggaggtacatgtcgctgttg
Glast	gagcctcaccaaggaagatg	cctcccggtagctcatttta

HPRT: hypoxanthine-guanine phosphoribosyltransferase; AQP: aquaporin.

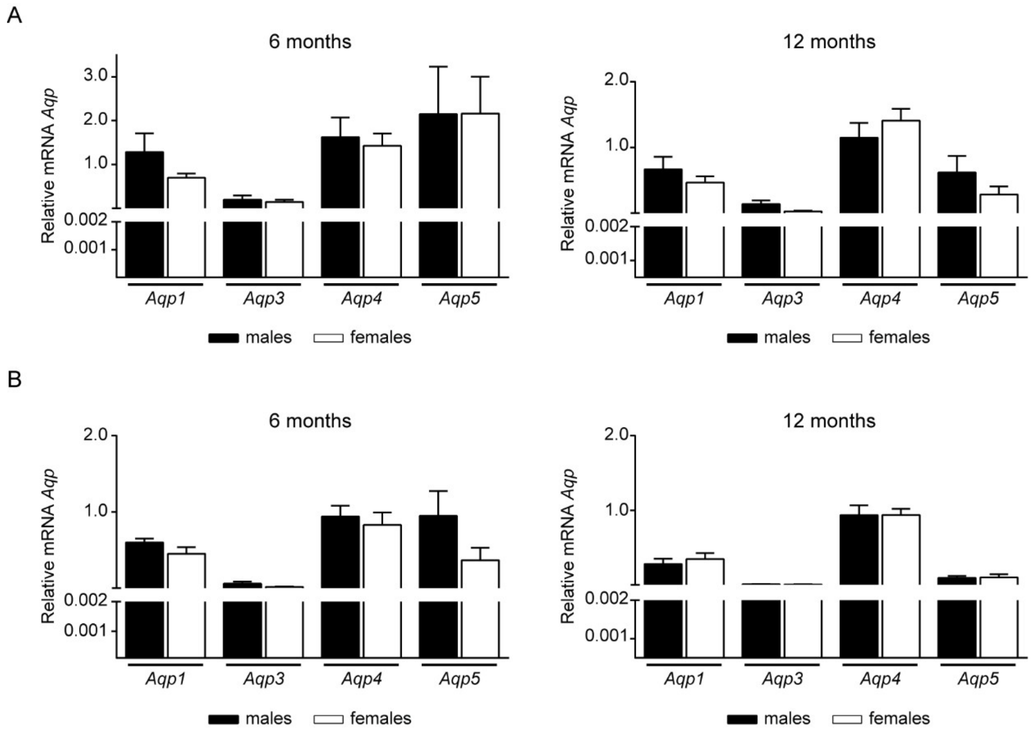

Supplementary Figure S1. RT-PCR analysis of the differential gene expression of *Aqps* in retinal samples derived from 6- and 12-month-old female and male APP/PS1 (**A**) and wt (**B**) mice. Data expressed as media ± SEM. * $p < 0.05$, *** $p < 0.001$, n = 12 (6 male and 6 female) mice per group, using two-way ANOVA and Bonferroni's multiple comparison post-test. wt: wild type.
